# An Automated In-Depth Feature Learning Algorithm for Breast Abnormality Prognosis and Robust Characterization from Mammography Images Using Deep Transfer Learning

**DOI:** 10.3390/biology10090859

**Published:** 2021-09-02

**Authors:** Tariq Mahmood, Jianqiang Li, Yan Pei, Faheem Akhtar

**Affiliations:** 1 The School of Software Engineering, Beijing University of Technology, Beijing 100024, China; tmsherazi@ue.edu.pk (T.M.); lijianqiang@bjut.edu.cn (J.L.); 2 Division of Science and Technology, University of Education, Lahore 54000, Pakistan; 3 Beijing Engineering Research Center for IoT Software and Systems, Beijing 100124, China; 4 Computer Science Division, University of Aizu, Aizuwakamatsu 965-8580, Japan; 5 Department of Computer Science, Sukkur IBA University, Sukkur 65200, Pakistan; fahim.akhtar@iba-suk.edu.pk

**Keywords:** breast cancer mass, deep learning, mammography classification, deep transfer learning, augmentation, computer-aided diagnosis

## Abstract

**Simple Summary:**

Diagnosing breast cancer masses and calcification clusters is crucial in mammography, which reduces disease consequences and initiates treatment at an early stage. A misinterpretation of mammography may lead to an unneeded biopsy of the false-positive results, decreasing the patient’s chances of survival. This study aims to increase the probability of early breast mass identification to ensure better treatment and minimize mortality risk. However, this study proposes a deep learning method based on convolutional neural networks to extract features of varying densities and classify normal and suspicious mammography regions. Two different experiments were carried out to validate the consistency of diagnoses and classification. The first experiment consisted of five end-to-end pre-trained and fine-tuned deep convolution neural networks. Additionally, the deep features extracted are used to train the support vector machine algorithm, resulting in an outstanding performance in the second experiment. Furthermore, this study confirms an improvement in mass recognition accuracy through data cleaning, preprocessing, and augmentation. Our deep learning hybrid model obtained a classification accuracy of 97.8%, outperforming the current state-of-the-art approaches. The proposed model’s improvements are appropriated in conventional pathological practices that conceivably reduce the pathologist’s strain in predicting clinical outcomes by analyzing patients’ mammography images.

**Abstract:**

Background: Diagnosing breast cancer masses and calcification clusters have paramount significance in mammography, which aids in mitigating the disease’s complexities and curing it at early stages. However, a wrong mammogram interpretation may lead to an unnecessary biopsy of the false-positive findings, which reduces the patient’s survival chances. Consequently, approaches that learn to discern breast masses can reduce the number of misconceptions and incorrect diagnoses. Conventionally used classification models focus on feature extraction techniques specific to a particular problem based on domain information. Deep learning strategies are becoming promising alternatives to solve the many challenges of feature-based approaches. Methods: This study introduces a convolutional neural network (ConvNet)-based deep learning method to extract features at varying densities and discern mammography’s normal and suspected regions. Two different experiments were carried out to make an accurate diagnosis and classification. The first experiment consisted of five end-to-end pre-trained and fine-tuned deep convolution neural networks (DCNN). The in-depth features extracted from the ConvNet are also used to train the support vector machine algorithm to achieve excellent performance in the second experiment. Additionally, DCNN is the most frequently used image interpretation and classification method, including VGGNet, GoogLeNet, MobileNet, ResNet, and DenseNet. Moreover, this study pertains to data cleaning, preprocessing, and data augmentation, and improving mass recognition accuracy. The efficacy of all models is evaluated by training and testing three mammography datasets and has exhibited remarkable results. Results: Our deep learning ConvNet+SVM model obtained a discriminative training accuracy of 97.7% and validating accuracy of 97.8%, contrary to this, VGGNet16 method yielded 90.2%, 93.5% for VGGNet19, 63.4% for GoogLeNet, 82.9% for MobileNetV2, 75.1% for ResNet50, and 72.9% for DenseNet121. Conclusions: The proposed model’s improvement and validation are appropriated in conventional pathological practices that conceivably reduce the pathologist’s strain in predicting clinical outcomes by analyzing patients’ mammography images.

## 1. Introduction

Breast cancer has an extremely high incidence in women and is leading cause of mortality, and its occurrence is increasing throughout the globe compared to other cancers [1]. Early diagnosis of breast anomalies by imaging is critical for maximizing the survival rate of breast cancer patients treated aggressively. Medical image interpretation for breast cancer risk assessment, screening, prediction, and treatment is becoming more significant. However, diagnosing malignant masses is time-consuming and challenging for radiologists to rule out the best treatment potential [2]. Medical imaging modalities such as magnetic resonance imaging (MRI), ultrasound (ULS), and mammography are accessible. The mammography images have become the first choice for breast masses screening, density measuring, and heterogeneity pattern recognition [3]. Daily increases in the number of mammograms raises the radiologist’s burden causing an increase in the misdiagnosis rate [4]. However, no matter the skills of doctors examining mammography, external factors such as image noise, fatigue, abstractions, and human delusion needs to be overcome, as the rate of misdiagnoses of breast masses during early mammography screenings are higher than 30% [5]. Furthermore, the scarcity of radiologists and their inconsistent allocation of resources are significant challenges that need to be overcome, particularly in developing countries. Additionally, the mammogram datasets are highly unbalanced and consist of a small number of images. Mammogram erroneous interpretations by doctors lead to conclusively harmful decisions to patients because breast biopsies are often advised if the diagnosis is malignant. However, 40–60% of biopsies are diagnosed as benign, distinctly revealing the need for accurate mammography examination to avoid needless surgeries, stress, and anxiety for the patients [6]. Finally, the implementation of deep learning schemes can help minimize incorrect interpretations and improve mammogram screening accuracy.

However, several deep learning-based (DL) methods, particularly convolutional neural network (CNN), have recently made remarkable achievements in various domains, including brain tumor prediction [7], skin tumor analysis [8], and breast cancer diagnosing [9]. The most widely used deep learning technique is CNN, which enables automatic mass recognition, feature learning and classification, applying smaller training datasets without human intervention. CNNs are constructed as a layer hierarchy [10]. Each layer converts input images to abstract images composed of edges, noise, and objects, and the final layer performs predictions using the pooled features [11]. Although the CNN model faces training issues due to the scarcity of labeled images, the manual categorization of the mammography is complex and prone to bias. CNNs sustain the mammogram images’ spatial integrity, such as how pixels are linked together to generate a distinct feature. Many CNN designs are available, including VGGNet, ResNet, GoogLeNet, MobileNet, and DenseNet, each of which has a distinct design that is optimized for various classification tasks. Deep convolutional neural networks (DCNNs) integrated with transfer learning concepts are utilized to effectively diagnose the suspicious areas in the mammogram, boosting radiologists’ screening performance. Transfer learning is an extensively used deep learning technique for predicting and interpreting breast mass, in which pre-trained models are retrained for a particular classification task [12,13]. The transfer learning (TL) methodology is initially trained on the ImageNet dataset, which can be used for generic feature extraction without additional training by modifying the architecture and hyperparameters. Model fine-tuning using TL is significantly more accessible and efficient than training from scratch with dynamically initialized weights. Recently, TL approaches have garnered tremendous interest and have made significant contributions to resolving feature extraction and classification concerns.

**Figure 1:**
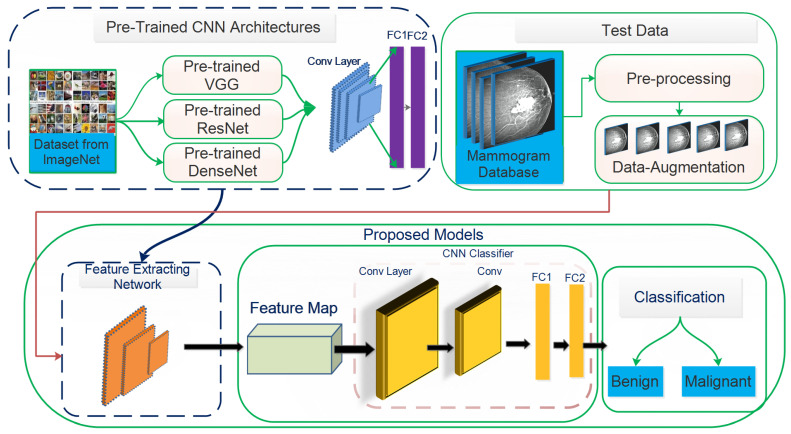
Step wise illustration of the transfer learning for deep CNN-based architectures, which contain blocks. (1) DCNN models are pre-trained on real images from Image-Net and used as feature extractors. (2) The data are pre-processed with a median filter to remove noise, then CLAHE enhances the images. (3) Data augmentation increases the number of the data samples, and (4) fine-tuning to share the properties of the DCNN model by transfer learning. (5) The final prediction is obtained by modifying the weights of the lower layer of pre-trained models to extract general features and fine-tuning the higher layer using global average pooling to attain a particular classification.

This study aims to increase the probability of early breast cancer identification to ensure better treatment and minimize the risk of mortality from breast cancer. However, this study proposes a fully automated deep learning-based method for recognizing, localizing, and classifying breast masses as benign or malignant under various imaging stipulations without expert involvement. Consequently, a hybrid model based on a convolutional neural network coupled with the support vector machines (ConvNet+SVM) has been proposed to learn and classify mammography features. Transfer learning is used to fine-tune the deep learning models to learn their effectiveness in specific clinical circumstances. The designed method’s results are compared with proposed end-to-end pre-trained deep learning algorithms. The proposed model’s whole architecture is described in Figure 1. In the proposed work, pre-trained DCNN models are fine-tuned for realistic breast mass categorization. Thus, we modified the pre-trained model’s architectures such as VGGNet16 [14], VGGNet19, MobileNetV2 [15], GoogLeNet [16], ResNet50 [17], DenseNet121 [18], and fine-tuned the final layers of every pre-trained model applying the TL approach to suit the problem. Every pre-trained model is trained partially by training specific layers while freezing the rest of the layers. In this situation, the network’s lower layers’ weights were left intact while retraining the model’s higher layers. The presented research demonstrates how TL can provide precise and consistent results when pre-trained models are used. Configurations of different models are studies to review breast masses to determine which proposed framework is best for breast masses diagnosis.

This study’s motivation is to help the radiologist enhance the fast and precise recognition rate of breast lesions using deep learning approaches and compare it with the manual system, which is time-consuming. The suggested approach can be summarized as:

- Initially, we preprocessed the obtained raw datasets using several preprocessing approaches and classified them into training and validation sets.
- We used pre-trained architecture including VGGNet16, VGGNet19, ResNet50, GoogLeNet, MobileNetV2, and DenseNet121 by fine-tuning the network’s final layers.
- We replaced the last pooling layer in each model’s last block with the global average pooling (GAP) and linked batch normalization layer (BN) with the GAP layer followed by the FC1, FC2, and output layers.
- We developed a hybrid deep ConvNet+SVM hybrid network to aid in the successful identification of breast cancer patients.

The rest of the work is structured into the followings sections: Section 2 is devoted to a review of the existing literature on mammogram-detected breast cancers. The background of deep learning techniques, transfer learning, and pre-trained neural network architecture are briefly discussed in Section 3. The proposed methodology for classifying breast cancer masses and image preprocessing methods are presented in Section 4. Section 5 describes different feature evaluation parameters. The experimental finding of the proposed work using various performance parameters is compared and shown in Section 6. Section 7 presents the discussion on the experimental findings. Finally, Section 8 summarizes the study’s results and suggests further research.

## 2. Related Works

Various deep learning-based algorithms have been designed to classify breast cancer masses in mammography images, which is the scope of this research. To mitigate the significant factors contributing to conventional machine learning (ML) approaches, deep learning methodologies have been proposed to extract relevant information and perform efficient classification tasks. The medical image modalities are integrated with deep learning approaches, improving the diagnosing ability of benign and malignant breast lesions. Features are extracted with the general-purpose learning method’s assistance in a deep learning system instead of being adjusted manually. DCNN has shown exceptional effectiveness in medical image processing, including lesion recognition, segmentation, detection, and quantitative analysis of breast masses in screening mammography images. Agarwal et al. [19] introduced a CNN-based automated system for mammography breast mass detection that integrates transfer learning and pertained models such as inceptionV3, VGG16, and ResNet50. The suggested CNN model learned feature using a CBIS-DDSM (curated breast imaging subset of DDSM) dataset, validated on the INbreast dataset. The InceptionV3 model performed admirably in classifying lesions, with a true-positive rate of (0.98±0.02) and a false-positive rate of 1.67 per image using the INbreast dataset. Samala et al. [20] presented a deep learning method based on transfer learning for feature extraction of breast anomalies, yielding excellent performance compared to the analytically derived characteristics. Shen et al. [21] exhibited a deep learning algorithm for mammographic images to detect breast masses. The proposed system used two pre-trained DCNN architectures, VGG16 and Resnet50, to identify the lesions. The designed model achieved an AUC of 88% on the CBIS-DDSM database and an average AUC of 95% using the INbreast dataset. The authors fused the best four models into an ensemble model to improve model reliability by applying the mean of their enhanced prediction values. The ensemble approach achieved the area under the curve ROC value by 91% for the CIBS-DDSM dataset and 98% for the INbreast dataset. Huynh et al. [22] used transfer learning approaches to extricate the spatial information from breast tumors, yielding better results than analytically extracted features. Al-antari et al. [23] proposed a deep learning-based method to segment and classify mammography images. The suggested system detects breast mass using the You-Only-Look-Once (YOLO) method and segments the identified masses using a full-resolution convolutional network (FrCN). Finally, to characterize the segmented lesion, a DCNN pre-trained AlexNet model with Adam optimizer and learning rate $lr^{-3}$ was used. The designed method achieved 95.64% classification precision and an AUC of 94.78%.

Almasni et al. [24] introduced a deep learning-based technique (YOLO) for identifying and classifying breast cancer masses. The proposed system performed preprocessing and feature extraction using a fully connected neural network (FC-NNS). The model’s performance was measured using the initial 600 images and 2400 augmented DDSM dataset, which yielded 97% accuracy and 96.45% AUC. Arora et al. [12] proposed a deep ensemble transfer learning-based method for feature learning and classification of breast masses. The CBIS-DDSM dataset was used to validate the model’s effectiveness and obtained 88% accuracy with an AUC of 88%. The author used adaptive histogram equalization to denoise the image, extract the valuable attributes, and identify them using a neural network. Shu et al. [25] developed a deep CNN classification model based on two pooling structures rather than the conventional pooling methods. The proposed approach consists of three steps: the feature extraction phase for feature learning and the pooling structures phase used to divide mammograms into regions with a high probability of malignancy based on the extracted features. The DenseNet169 model is used as the feature learner by modifying its final layer according to the pooling structure to classify the extracted feature. The model test on the INbreast database achieved 92.2% accuracy with an AUC of 92.4% and with CBIS-DDSM database attained 76.7% accuracy with an AUC of 82.3%. Ribli et al. [26] suggested an automated approach for identifying and diagnosing breast masses by utilizing the transfer learning methodology to incorporate a quicker RCNN model. They assessed the model’s performance using the INbreast database and obtained 95% AUC. Singh et al. [27] developed a conditional generative adversarial network to classify breast masses into ROI using mammogram images. The generative network determines how to build a binary mask that characterizes it to detect the tumor region. Additionally, a CNN-based shape descriptor is proposed for classifying the generated masks as irregular, lobular, oval, or round shapes. The suggested shape descriptor was trained using the DDSM database, achieving 80% accuracy. Dhungel et al. [28] proposed a computer-aided detection (CAD) method to detect, segment, and classify breast cancer masses. A deep belief network (m-DBN)-based approach is proposed for breast mass detection and a Gaussian mixture model (GMM) for ROIs extraction. Bayesian optimization is used to optimize predictions. Deep hierarchical output learning is used to segment and refine the detected ROIs. Finally, a pre-trained deep learning classifier was used for breast mass categorization, which achieved an average of 91% accuracy and 76% AUC on the INbreast dataset.

In the presented framework, transfer learning has been exploited to overcome existing systems’ deficiencies in detecting and classifying breast cancer masses and calcification. The dense breast’s clinical symptoms are not entirely clear; therefore, it is challenging to discern dense lesion features and perform lesion classification accurately. Furthermore, feature extraction is error-prone and time-consuming, increasing doctors’ responsibility; hence the proposed study provides a robust deep learning framework for breast mass diagnosis and classification. Currently, deep learning-based technologies are not designed to replace skilled physicians in clinical diagnosis; instead, they are intended to assist doctors in clinical decision-making. This study presents a model for automatically identifying breast abnormalities based on deep learning and convolutional neural networks. The presented approach applies a deep transfer learning model to extract features from the mammography images that automatically categorize breast cancer and determine whether it is malignant.

## 3. Background of Deep Learning Methods

Deep convolutional neural networks (DCNN) have been preferred in different research realms due to their promising biomedical image analysis and classification performance. Additionally, biomedical images include extraneous data, annotating information, and various markings that negatively affect automated image analysis methods. Deep learning algorithms help ensure a fast and precise breast cancer diagnosis that meets a credible radiologist’s preciseness. However, different low-level features are excerpted distinctly by the well-known CNN architectures of VGGNet (Visual Geometry Group Network) [14], MobileNet [15], GoogLeNet [16], ResNet (Residual Networks) [17], and DenseNet [18] in the proposed framework.

### 3.1. Feature Learning Using Convolutional Neural Network

LeCun et al. [29] applied CNN for the first time to recognize handwritten zip codes. In comparison to its progenitors, CNN’s key benefit is its ability to identify essential features without human interaction. The CNN model consists primarily of convolutional layers, a ReLU (nonlinear activation) layer, a pooling layer, a flattening layer, and a fully connected (FC) layer. CNN architecture uses various detectors (filters) such as edge and corner detectors to recognize the different objects by their shape, size and interpret images [30]. The convolutional layer aims to derive high-level features from the input image, such as edges and corners, and map these features to the subsequent convolution layer. The nonlinearity layer (activation) integrates a deep network into a nonlinear structure that quickly ascertains this layer. The pooling layer is added after the activation layer. Its core objective is to decrease the input image’s size (width × height) by retaining critical features within the feature mappings. The flattening layer collects information for the FC layer by reconstructing the convolution and pooling layer patterns to single-dimension information. The FC layer performs recognition and classification using the Softmax activation function to normalize the output. The 2D convolution process is elaborated in Equation (1).

(1) $$P\left(a,b\right)=\left(I\times K\right)\times \left(a,b\right)=\sum_{x}\sum_{y}I\left(a,b\right)\times K\left(a-x,\;b-y\right)$$

Here, *I* is the 2D image, *P* the output image, *a* and *b* the filtering location on the image during convolution process, *K* the filer matrix shifted on the image, *x* and *y* the filter position. The deep learning loss function (cost function) minimizes the variation within predicted and empirical output by attaining the optimized value for weights. For this purpose, various iterations with varying weights are processed. Gradient descent is an iterative ML optimization method used to decrease the cost function, enabling neural networks to obtain more precise judgments. During the training phase, the CNN employs adaptive delta (Adadelta) [31] and Stochastic Gradient Descent (SGD) [32] optimizers to update a prediction error as input and backpropagate the error to the network. Then, it is subsequently used to enhance filters in the convolution layers and weights FC layer [33]. The SGD optimizer updates all training samples within the database by exerting the learning and momentum coefficients consistently. The Adadelta learner does not update the learning coefficient every step. Apart from Adadelta, the Adam optimizer updates all training and momentum wights and network parameters. In order to prevent rapid gradient descent, the Root Mean Square Propagation (RMSProp) optimizer adjusts the learning coefficient to minimize the effect of exponential regression [34].

### 3.2. Transfer Learning

Transfer learning is a technique that uses a trained model for one task as the starting point for solving other tasks [35]. Thus, pre-trained methods are preliminary for particular tasks in transfer learning, rather than going through a lengthy training procedure with randomly initialized weights. Pan and Yang [36] proposed a method for accurately understanding transfer learning by combining domain, task, and marginal probabilities. The domain *D* was described as a tuple of pair elements composed of feature space, χ with marginal possibility, and *K(S)* a sample data point. Thus, domain *D* stated mathematically as in Equation (2).

(2) $$\begin{array}{c} D=\{\chi ,K(S)\} \end{array}$$

where, *S* is a particular learning sample $S=s_{1}\ldots ,s_{n}.\varepsilon \chi$, χ each term vectors space, $s_{i}$ is the $i^{th}$ term vector resembling any texts. Hence, it reduces the significant computational resources required to build neural network models to resolve such difficulties.

(3) $$\begin{array}{c} T=\{\gamma ,K\left(R|S\right\}=\left\{\gamma ,\eta \right\}\times \left\{\gamma ,\eta \right\},R=\left\{r_{1}\cdots ,r_{n}.\right\},r_{i}\times \varepsilon \gamma \end{array}$$

Equation (3) illustrates task *T* for domain *D*, γ label space, η predictive function that extracts features from $\left(s_{i},s_{i}\varepsilon \chi \right)$ and $\left(r_{i},r_{i}\varepsilon \gamma \right)$.

(4) $$\begin{array}{c} \gamma \left(s_{i}\right)=r_{i} \end{array}$$

γ in Equation (4) is a predicting a label for all feature vectors. Training deep learning models for medical diagnosis-related problems is becoming more difficult due to small datasets with unsatisfactory performance. Consequently, a deep learning methodology is trained on huge datasets. A model is taught from prior information and applied to one issue before being reused for subsequent problems, allowing for the rapid development of accurate models. Therefore, the presented architectures produced credible results since the weights were trained on the ImageNet dataset and can learn generic features from other datasets without requiring further training. Additionally, each of these pre-trained methods has been fine-tuned to classify the breast masses or calcification. Each layer of the architecture was more trainable. The transfer learning method requires selecting pre-trained models based on associated target issues, issue size, and similarity. An overfitting issue will increase if the target dataset is less than the source dataset (less than 1000 images). If the target dataset is large, fine-tuning the model is necessary to prevent an overfitting problem. A simplistic operation achieves higher performance by adding a new FC layer(s) into pre-trained models. Features derived independently from the particular CNN design are fused into the fully connected layer to classify breast masses into normal and abnormal using average pooling classification.

### 3.3. Pre-Trained Neural Networks

Various transfer learning approaches have recently been used for screening and interpreting biological images. Currently, six (VGGNet16, VGGNet19, MobileNetV2, GoogLeNet, ResNet50, and DenseNet121) deep CNN architectures share their transfer learning and tuning characteristics. All CNN architectures adopted in the proposed study were trained on ImageNet by sample images, and transfer learning has been applied. Fine-tuning allows the architecture to learn generic characteristics automatically from other datasets without further training. The fine-tuned features are combined and fed into an FC layer to classify the breast masses or calcification. Fused features may hold several characteristics derived from shape descriptors (compactness, roundness, circularity, etc.).

#### 3.3.1. VGGNet16

Simonyan and Zisserman [14] suggested the VGGNet16 model, commonly used in different disciplines due to its remarkable adaptation abilities and comparatively simple layout. The VGGNet16 model achieves the highest testing accuracy of 92.7% on ImageNet, a database with over 14 million images, including 1000 classes. It contains 16 layers, so the model is called VGGNet16. VGGNet16 is comprised of thirteen convolution layers, ReLU, pooling, and three FC layers. The pre-trained VGGNet16 composes five blocks, and each max-pooling layer has a variable degree of specificity in the in-depth information. The lightweight layer preserves local patterns while the deep layer obtains global patterns. The VGGNet16 architecture takes RGB images of dimensions of 224×224 with a 3×3 filter size for the convolution network and 2×2 filter size for the pooling network, both with a stride of 1. There are three FC layers in the final section, each with 4096,4096, and 1000 neurons. The final layer is the Softmax, which generates a statistical value of 0 or 1, representing the node’s output class. The ReLU layer serves as an activation function in every hidden layer.

(5) $$\begin{array}{c} K(x)=max(0,x) \end{array}$$

**Figure 2:**
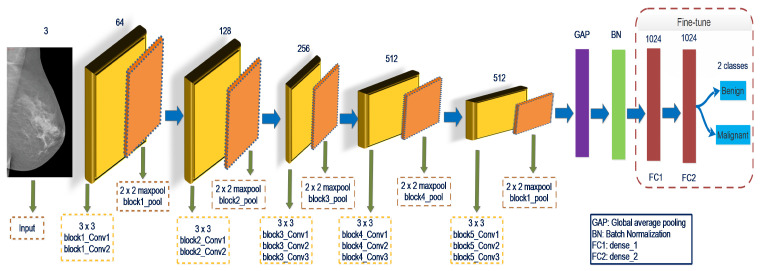
Representation of the network structure of the VGGNet16 model composed of 16 layers. The global average pooling layers (GAP) and batch normalization layer (BN) are added to obtained the global information and succeeded by FC1, FC2, and output layers. The weight is optimized to learn for the specific tasks during model training.

In Equation (5), *x* is less than zero, K(x) is zero. If *x* is greater than or equal to zero, then K(x) equals *x*. Local response normalization (LRN) is not used in the VGGNet16 network; this normalization method does not enhance performance in the ImageNet dataset but increases resource usage and computation cost [37]. The VGGNet16 network contains approximately 138 million trainable parameters. Fine-tuning of the final layers shown in Figure 2 will improve the characterization learning of the input data and increase the ability of the classification method. The particular strategy is to execute the GAP layer on the pooling layer of the final block. Batch normalization will be connected to the GAP layer, followed by the FC1 and FC2 layers, and finally, the output layer. Although the convolutional layers’ weights are fixed throughout model training, the FC1, FC2, and output layers’ weights are fine-tuned to collect domain-specific information.

#### 3.3.2. VGGNet19

**Figure 3:**
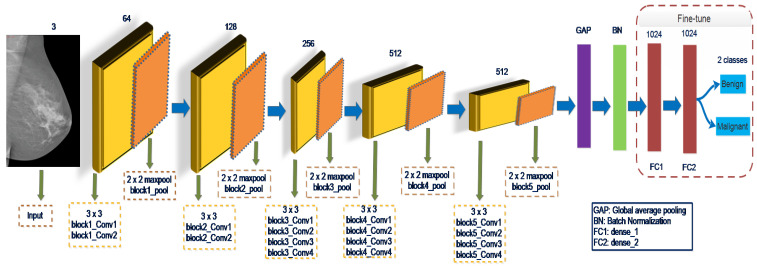
Representation of the network structure of the VGGNet19 model containing 19 layers. The global average pooling layers (GAP) and batch normalization layer (BN) are added and succeeded by FC1, FC2, and output layers. The weight is optimized to learn for the specific tasks during model training.

Simonyon and Zisserman [14] suggest a VGGNet19 network contains three additional convolution layers than VGGNet16, in which sixteen are convolutional while the other three are fully connected layers. Convolution layers of stride 1 use 3×3 size filters. Max-pooling activities are performed using a 2×2 window scale and a stride of 2. Each of the three FC layers contains 4096, 4096, and 1000 neurons. Softmax is the final layer, and ReLU operated as the activation function for all hidden layers. VGGNet19 architecture is devoid of LRN and contains approximately 143 million in learnable parameters [37]. The fine-tuning of the last layers of VGGNet19 shown in Figure 3 will improve the input data’s characterization learning and increase the classification model’s capability. The GAP and BN layers are added to the last block’s pooling layer. Following the GAP layer, the batch normalization layer, the FC1, FC2, and output layers will be connected. The weight is optimized to learn for the specific tasks during model training.

#### 3.3.3. GoogLeNet

The GoogLeNet framework is a much deeper and broader framework trained on over a million images and classifies them into 1000 classes. It has 22 layers with different inception modules with differing filter sizes related to the inputs and integrating the results. The multiscale processing enables the system to disengage characteristics in distinct levels concurrently. GoogLeNet impersonates the thought of adopting a GAP layer rather than fully connector layers, limiting the number of model parameters. Using the network of GoogLeNet, we suggested a model that fuses different convolution filters of distinct sizes into a unique novel filter, decreasing the occurrence of perimeters and computing complexity. The GoogLeNet has few convolution parameters in the network’s core and employs the GAP layer at the model’s end rather than FC layers. The inception module works in parallel with the convolutional and pooling layer, enabling several features to capture simultaneously. Ultimately, a filter concatenation layer integrates the results of every parallel layer. Our research used 09 inception modules architecture, where the final layer is modified with fine-tuning with GAP and BN layer to satisfy the classification problems. The last final layers are liable for specifying the accurate classes to the input images. However, the FC layer is described by the number of classes correlated with the network’s last migrated layer to different layers.

#### 3.3.4. MobileNetV2

MobileNetV2 is a low-power model developed in collaboration with a Google community. It is an improvement over MobileNetV1 that employs depth-wise separable convolutions as effective key components. The structure contains residual blocks with a stride of one, and downsizing blocks have two. Aside from that, it has longitudinal bottlenecks between the layers, which is important because it avoids non-linearity from destroying an excessive amount of information. These bottlenecks facilitate the model’s encoding of intermediary input and outcomes. The hidden layer aids in the transformation of lower-level meanings like pixels to higher-level identifiers. Additionally, there are shortcut relations between the bottlenecks. This study enhances the model performance by modifying the last layer of the final block with GAP and BN layers using transfer learning. The last layers of the model (FC1, FC2, and output layers) are liable for specifying the accurate classes to the input images.

#### 3.3.5. ResNet50

**Figure 4:**
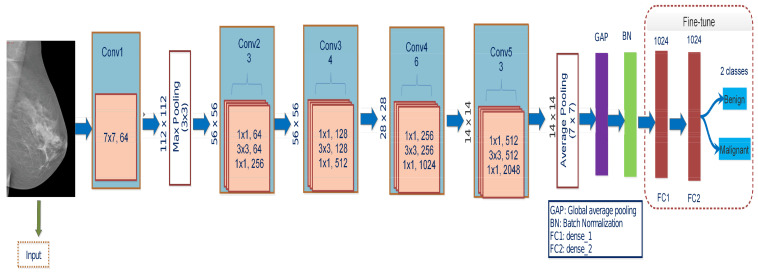
The proposed framework of ResNet50 architecture containing 50 layers. The global average pooling layers (GAP) and batch normalization layer (BN) are added, followed by FC1, FC2, and output layers.

The residual networks, called ResNet, have a different architecture than the typical sequence CNN model. With the various connections used, ResNet seeks to overcome the deterioration issue in CNN networks. The deterioration issue happens when deep networks tend to converge [17]. ResNet50 combines multiple-sized convolution filters to manage the degradation problem and conquer training time due to deep structures. The architecture is redesigned by fine-tuning final block layers shown in Figure 4, substituting the top layers with GAP layer, BN layer, FC1, FC2 layers, and the final layer with a Softmax, which allows us to recognize two diagnostic classes. The input images’ sizes are all resized to 224×224 compatible with this model. During training, the Adam optimizer is used, which has a learning rate of $Lr^{-3}$. It uses the identity function to map parameters with no parameters and adds the last layer’s output to the preceding layer. The identity mapping of a shortcut channel is multiplied by a linear projection to accommodate the residual.

#### 3.3.6. DenseNet121

From the literature, the connections between layers near the input and output layers contribute to the efficacy of convolutional networks. This concept has been applied in ResNet, and dense convolutional networks [18]. DenseNet uses a basic connectivity pattern to validate the flow of knowledge within layers in forwarding and backward computation to learn the vanishing gradient problem. This study proposed a densely connected DenseNet neural network model with a couple of characteristics. Each layer is linked to the preceding layer to reuse features. The DenseNet structure consists of the DenseBlock layer, containing 6, 12, 24, and 16 convolution blocks, and the Transition layer. In the whole DenseBlock, each layer’s output with k-characteristic is mapping after convolution. The convolution layers are responsible for feature extraction, avoiding the manual feature extracting’s misdiagnosing. Each layer uses the previous layer’s feature maps as its input, and each layer’s feature maps from the subsequent layers transfer all the data by explicitly linking all the layers in the network. DenseNet concatenates the previous layer’s output with the output of the succeeding layer. Traditional feedforward neural networks combine the $i^{th}$ layer findings to the succeeding layer ${\left(i+1\right)}^{th}$ by applying the composite function. The corresponding elements are accumulated and then passed to a ReLU function to achieve the feature mapping and extraction. These methods include convolution, activation, pooling, and batch normalization. Equation (6) represents the above operations:

(6) $$\begin{array}{c} a_{i}=F_{i}.\left(a_{i-1}\right) \end{array}$$

here $F_{i}\left(.\right)$ denotes a nonlinear transformation function, a combination that includes a series of BN, ReLU (nonlinear function), pooling, and convolution operations. The ResNet architecture is extends Equation (6) by using shortcut connections as demonstrated in Equation (7)

(7) $$\begin{array}{c} a_{i}=F_{i}.\left(a_{i-1}\right)+\left(a_{i-1}\right) \end{array}$$

DenseNet concatenates the layer’s output feature maps with the incoming feature maps rather than sum them. Thus for DenseNet Equations (6) and (7) vary as illustrated in Equation (8). The transition layer attaches two nearby DenseBlock layers and limits the features mapping dimension. The structure of the transition layer, including a convolution layer (1×1), the pooling layer (2×2), and its architecture is BN+ReLU+Conv (1×1) + pooling layer (2×2) that function as a compression. Each layer in the transition layer is attached to all previous layers as input.

(8) $$\begin{array}{c} a_{i}=F_{i}.\left(\left[a_{0},a_{1},\ldots ,a_{i-1}\right]\right) \end{array}$$

The ultimate DenseBlock is followed by a GAP layer transferred to a Softmax classifier for image identification and classification. The feature maps of every previous layer $a_{0}$, and $a_{i-1}$, are received as input by $i_{th}$ layer. Since Densenet121 concatenates feature maps, the dimension of the channel in all layers is increased. If $F_{l}$ is applied to create *S* function maps, then generalization in Equation (9) may be applied to the $i_{th}$ layer.

(9) $$\begin{array}{c} i_{l}=S_{0}+S\times \left(i-1\right)) \end{array}$$

**Figure 5:**
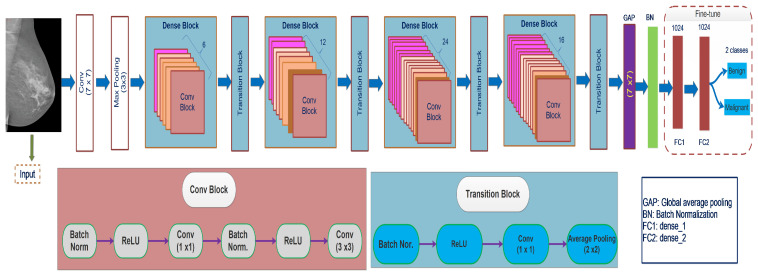
Representation of the network structure of the DenseNet121 model.

$i_{l}$ represents the input feature maps, $S_{0}$ the input channels, and the *S* parameter growth rate. The growth rate controls the amount of data appended to the system at every layer. Feature maps are the network’s details, and each layer has connects to previously functioning maps to integrate data. Figure 5 represents the DenseNet121 architecture connection mechanism.

### 3.4. Feature Classification Using Support Vector Machine (SVM)

SVM is a machine learning method based on the spatial risk mitigation concept that is highly effective in pattern recognition, inferential analysis, time-series analysis, and other realms [9]. However, it performs better in classifying mammography lesions as benign or malignant based on their features with fewer computations. The objective of SVM classifiers is to construct a hyperplane to create an efficient method for diagnosing and classifying mammogram images. It is frequently used with insufficient training datasets to achieve more significant generalization and overcome linear and non-linear to separate data points of each class. It aims to mitigate the percentage of nonzero weights and the overfitting problems. Recently, classification using the convolutional neural network with the support vector machine (ConvNet+SVM) method has gained popularity.

The proposed study has heightened prediction accuracy with SVM as the top layer in deep learning by modifying the Softmax layer. Using the obtained mammography image dataset, we observed that the SVM model marginally outperforms the DCNN model for mass breast identification. The rationale of this improvement is obscure; it may be solely due to a better optimization method or may be associated with the dataset’s diversity and non-linear nature. It may be because the SVM advances toward a global minimum and tolerates more noise (variation in the patterns linking to the original images), making it imperceptibly more robust to an extensive collection of features. Consequently, SVM may have remarkable convergence and robustness benefits over DCNN models.

**Figure 6:**
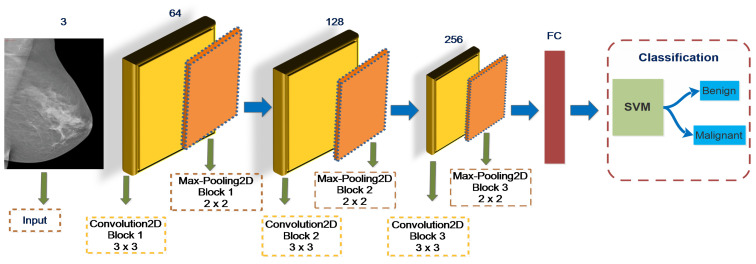
Features extraction from fully connected layer to be input into SVM classifier.

This study proposes end-to-end DCNN architectures including VGGNet16, VGGNet19, GoogLeNet, ResNet50, MobileNetV2, and DenseNet121 to identify breast cancer lesions. The experimental findings reveal that the SVM classifier is generally more successful than Softmax. The highest training accuracy rates are obtained 97.8% for ConvNet+SVM compared to 90.2% for VGGNet16+Softmax, 93.5% for VGGNet19+ Softmax, 63.4% for GoogLeNet+Softmax, 82.9% for MobileNetV2+Softmax, 75.1% for ResNet50+Softmax, and 72.9% for DenseNet121+Softmax. The architecture of ConvNet+SVM is depicted in Figure 6.

## 4. Materials and Methods

Breast cancer is the most prevalent kind of cancer and is associated with the highest cancer-related mortality in females. Screening mammograms for breast cancer is an effective way to detect the disease. Preprocessing of mammogram images included resizing, scrambling, and normalizing the data [27]. Initially, the image acquisition method is carried out where morphological opening and closing operations are used to eliminate annotation labels from images. Moreover, mammography noises, such as Gaussian, Salts and Pepper, Speckle, and Poisson noises are suppressed from the image using a Median, Gaussian, and Bilateral filtering technique. Additionally, the image’s contrast is increased by applying Contrast Limited Adaptive Histogram Equalization (CLAHE) method. The area around the breast mass is then segregated using the OSTU threshold method. This study increased the size of the datasets to almost eight times the core datasets volume using various data augmentation methods, including flipping, rotation, scaling, and brightness. The preprocessed database was separated into a training and testing set to train the ConvNet+SVM and CNN-pre-trained architectures using the training data.

This section presents the proposed framework based on ConvNet+SVM, ConvNet, and DCNN for detecting and classifying malignant breast tissues in mammographic images. Different low-level features are excerpted distinctly by well-known CNN architectures of VGGNet [14], MobileNet [15], GoogLeNet [16], ResNet [17], and DenseNet [18] in the proposed framework. However, this study aims to process automatic feature engineering and analyze transfer learning concepts on distinct deep learning architectures. The proposed model’s performance is enhanced by iterating over various training models with hyperparameter values. The suggested architecture is shown in Figure 1.

### 4.1. Preprocessing Mammography

The collected dataset comprises low-quality images containing many missing data, noises, and sizes that create high false-positive and negative rates. However, each image needs to be preprocessed (normalized and resized) in compliance with the deep neural network parameters [38]. This work uses screening methods focused on matching distinct areas using image filters and image enhancement methods. Image enhancement intensifies the visual characteristics includes margins, boundaries, and contrasts, and eliminates the artifacts. This study employed Median, Gaussian, and Bilateral filters to remove the different mammography noises. However, later the CLAHE technique was utilized to enhance the quality of mammography images [39]. Using the OTSU threshold, we extract the breast region from the context and exclude part of the breast region, including objects, labeling, and patient information from mammogram images. Using the OTSU threshold, we isolate the breast area from its environment and eliminate a part of the breast region including objects, labeling, and patient information from mammography images. Finally, the extraction of suspicious areas and feature matching are utilized to identify breast cancer masses or calcification without expert involvement. It attains excellent breast mass detection and classification performance with different shapes, edges, and anomalies. The extracted ROIs comprising the mass area were used to train and evaluate the proposed architectures to classify breast masses as benign or malignant.

#### 4.1.1. Mammogram Resizing

Different DCNN models needed input images of varying dimensions according to prescribed architecture. However, all collected images are resized into a fixed scale of 224×224 using nearest-neighbor insertion. Although the collected images are grayscale, the DCNN pre-trained models need RGB input images as colored images are used to train the models. Consequently, these images are transformed to input image RGB by mimicking the single channel to make a 3-channel RGB image. We used a 6346 data sample for model training and 913 for model testing based on the data splitting. We propose an automatic ROI segmentation technique that overcomes mass complexity, such as textures, regions, and unclear edges while achieving a satisfactory efficiency.

#### 4.1.2. Zero-Mean Normalization

The proposed study uses normalization based on the respective structures to address mammography disturbance from irregular illumination, speckle noise, undesirable formatting, and morphological adjustments [40]. Image normalization provokes the image to invent unusual invariants, intensifies robustness, and speeds up the training model’s confluence. The processed data complies with the standard normal distribution with mean 0, and the standard deviation (SD) is 1. The conversion function is declared in Equation (10)

(10) $$X^{*}=\frac{\left(x-\mu \right)}{\sigma }$$

here, μ represents the mean, and σ represents all sample data’s SD.

#### 4.1.3. Data Augmentation

The primary issue in the field of mammographic images is the lack of publicly available datasets. Although few datasets are available on the Internet, the number of images specifically for our problem is significantly less. Adequate training of a deep neural architecture needs an extensive amount of data. With the scarcity of mammography availability, model parameters are eroded, and trained systems perform inefficiently. Different data augmentation techniques address the data scarcity issues by making efficient use of current data. It increases the sample size of the current training data and prevents the model from overfitting.

Thus, we propose distinct data augmentation techniques for enhancement training samples comprising Gaussian scale-space (GST) theory and data amplification settings (scaling, cropping, rotating, shifting, flipping, and cropping). We build batches of tensor image data with real-time data augmentation using Keras’ ImageDataGenerator library [41]. It overcomes the overfitting problems and makes the system transformational and noise invariant. However, every mammography image in the benign and malignant cases in the dataset is expanded eight times. Consequently, the proposed dataset contains 2667 benign and 4592 malignant mammography images after data enhancement. The techniques and configurations applied for image data augmentation as described in Table 1.

### 4.2. Architecture Fine-Tuning

The preprocessed and normalized mammography images train the proposed architectures to classify predetermined breast masses or calcification as normal or malignant. This study improved the DCNN architectures by fine-tuning the pre-trained model (VGGNet16, VGGNet19, ResNet50, MobileNetV2, GoogLeNet, and DenseNet121) to discriminate the breast cancer masses or calcification. All layers of the networks were trainable that extricated the features from the mammography. However, this study fixed the weights of the lower layer of pre-trained models to extract the generic features while fine-tuning the higher layer with the GAP to perform the specific classification task. The suggested approach is implemented by changing every model’s last block’s final pooling layer with the global average pooling. The batch normalization layer will be linked by the GAP layer followed by the FC1, FC2, and output layers. The weights of the convolutional layers are configured using proposed pre-trained models, while the rest are initialized dynamically. The weights of convolutional layers are fixed during model testing, whereas FC1, FC2, and output layers are fine-tuned to extract field-relevant knowledge. The information flowing across the various channels will be strongly correlated. The convolution layer will merge cross-channel information, resulting in improved dimension reduction and efficient parameter reduction. Each model is trained for about 90 epochs applying an adaptive optimizer (Adam). The learning, momentum, and weight decay rates are all adjusted to 0.001, 0.9, and 0.0001, accordingly, as explained in Table 2. These configurations ensure the network fine-tuning by freezing the weights of particular layers to suit our classification task.

### 4.3. Class Activation Maps

This article identifies how the global average grouping is used to build a CNN activation map. Class activation mapping refers to the weighted activation mapping created for every image that demystifies the consequences of DL models. Traditionally, deep learning methods are often considered black-boxing. We use the pre-defined architectures consisting of convolutional layers and the final output layer (Softmax). A fully connected network is retained after the GAP layer, followed by the Softmax layer, which gives a class prediction. We implement GAP on the convolutional feature mapping and use these characteristics to produce the most performant, fully connected layer. The system needs to be trained using the GAP layer to obtain the class activation map. It assists in identifying the suspect region in mammography on which the model should concentrate before generating the final prediction, providing insight into how the algorithm works. It is critical to comprehend the results of the deep learning-based clinical decision-making model. Additionally, this research helps in hyperparameter tuning and identifying the underlying reason for the model’s failure. We compute the $A_{c}$, the class activation mapping for class *c*, in which each spatial variable is represented by

(11) $$A_{c}\left(a,b\right)=\sum_{x,y}w_{k}^{c}\times f_{k}\times \left(a,b\right)$$

In Equation (11), $A_{c}\left(a,b\right)$ represents the activation mapping for unit *k* with in final convolutional layer at a given spatial position (a, b). For unit *k*, GAP finding $f_{k}$ is $\sum_{x,y}f_{k}\left(x,y\right)$. However, class score for a given class *c* is $\sum_{x,y}w_{k}^{c}$, here $w_{k}^{c}$ represents the weight for unit *k* as per class *c*.

## 5. Feature Evaluation

The effectiveness of the proposed frameworks in obtaining mammography datasets is evaluated by modifying pre-trained models using transfer learning. Our proposed models’ classification performance was evaluated using Python’s sci-kit-learn module, which comprises accuracy, precision, recall, and F1-score. Various performance metrics may be computed as follow:

- TrP (True positive): is a positive instance accurately diagnosed as positive (malignant).
- FaN (False positive): a a positive instance is mistakenly interpreted as a negative (benign).
- TrN (True negative): is a negative instance diagnose as a negative (benign).
- FaP (False positive): is a negative instance mistakenly detected a positive (malignant).

*Accuracy* is the probability of perception that is diagnosed exactly correct from the whole observation. Equation (12) is used to determine the accuracy.

(12) $$Accuracy\left(ACC\right)=\frac{TrP+TrN}{TrP+FaN+TrN+FaP}\times 100\%$$

*Precision* is computed as the ratio of successfully diagnosed positive cases to the predicted cumulative number of positive cases. The term “higher precision” refers to the fact that mammography labeled as positive is indeed positive. Equation (13) is used to assess precision.

(13) $$Precision=\frac{TrP}{TrP+FaP}\times 100\%$$

*Recall* is defined as the probability of a positive diagnostic instance from the whole observation. It is the true-positive estimation of all true-positive and false-negative. High recall and low precision indicate that most positive cases are correctly identified, some are false-positive. Low recall and a high precision suggest that certain positive instances were missing, but only a few are correctly identified. Equation (14) is used to determine the recall.

(14) $$Recall=\frac{TrP}{TrP+FaN}\times 100\%$$

*F1-Score* provides an optimal combination between accuracy and recall. F-measure is computed applying Equation (15).

(15) $$F1=\frac{precision\times recall}{precision+recall}\times 2$$

We used the receiver operating characteristic curve (true-positive and false-positive rate) and its area under the curve (AUC) to determine the overall efficiency of our developed models.

## 6. Experimental Work Setup

This section depicts the examinations and evaluation criteria to validate the feasibility of the proposed models. The proposed architecture is fully automatic and capable of diagnosing and interpreting various mammography images without human intervention. Keras [42], an open-source deep learning library, is used in association with TensorFlow [43] as the backend for loading and fine-tuning the pre-trained architectures on the ImageNet database. Both experiments were conducted using a system equipped with an Intel Core i7 CPU, 8GB RAM, and an NVIDIA GTX 1060Ti graphics card, as well as the Keras-TensorFlow and MATLAB2017a environments.

### 6.1. Mammogram Dataset

The dataset consisted of 7259 mammography images and was divided into a training and test set. There were 6346 mammography images (87.4% of the dataset) in the training set and 913 (12.5% of the dataset) in the test set to validate the trained model’s accuracy. To train and evaluate the proposed techniques, 7259 mammography images from three databases were used. These images include benign and malignant masses or calcification of varied sizes, densities, forms, and margin patterns, as explained in Table 3. The dataset used in this analysis contained no instances of normal mammogram images. To assess the efficacy of the DCNN models used in this study, we obtained all mammography images from the standard benchmark Mammographic Image Analysis Society [44] (MIAS), INbreast [45], and a private hospital in Pakistan.

MIAS and INbreast datasets are publicly accessible and widely used for research. The private dataset was acquired with the approval of the Institutional Review Board (IRB) of Continental Medical College and Hayat Memorial Teaching Hospital, Lahore, Pakistan [46]. The new dataset consisting of digital mammography images is accessible upon request for research objectives. The proposed study was approved by the IRB, Continental Medical College, and Hayat Memorial Teaching Hospital, and consent has been obtained from the hospital for experimentation purposes without disclosing patients’ personal information due to privacy and ethical concerns. The radiologist team is comprised of two senior radiologists, both with experience of eighteen years in this field. Professional radiologists manually annotated/labeled the new mammogram images to classify the breast masses as benign or malignant.

The symmetry of the images distributed for learning and testing was highly imbalanced. Consequently, the databases were shunted and segregated into training and testing datasets only. The data augmentation approach is applied to obtained datasets by scaling, rotation, translation, and color modeling to generate 7259 mammographic images. However, the performance of the MIAS dataset is very vulnerable because of the minor variation in intensity between the microcalcification and its surrounding regions within mammography images.

### 6.2. Results Analysis in Term of Accuracy and Loss

Each experiment was conducted to assess the efficacy and performance of different configurations of pre-trained networks based on transfer learning for mammography classification. The model’s parameters and activation functions are fine-tuned through training to develop a practical framework for each model. To achieve accurate predictions, hyperparameter values are manually iterated to fine-tune and refine the conceptual DCNN architecture. Hyperparameters restrain the training algorithm’s behavior by manually setting the values of variable training parameters before starting training. The evaluation process has been carried out under similar training and validation settings for each DCNN model. The proposed ConvNet+SVM model’s findings are correlated to a fused feature set and other developed techniques. The experiments aim to obtain the best architecture for all pre-trained model that performs excellently in learning and testing databases.

Consequently, designing a DCNN model aims to diagnose breast masses accurately when being tasted on new databases. The data augmentation techniques are used to enhance the number of mammogram images used for model training. As represented in Table 3, a dataset including 2667 benign and 4592 malignant images is applied to training and validating the models. However, 6346 images, both benign and malignant, are included in the training phase. The training accuracy and loss curves were computed after 90 epochs of training models, each epoch corresponding to a complete route of the training algorithm within the training set. Initially, every model is trained by applying the Adam optimizer with hyperparameter values such as learning rater $Ie^{-4}$, batch size 32, and L2-Regularization. The experimental results are compared by targeting the high accuracy and low loss value, as illustrated in Table 4. The Adam optimizer using a learning rate of $1e^{-4}$ during training achieves high accuracy and fewer loss values and does not experience overfitting problems. The discrepancy between training and validation accuracy is 0.1% when the Adam optimizer is used. The model’s design has the lowest generalization difference between testing and validation losses at 0.0324. The linear kernel SVM classifier built with the deep function achieved the highest classification accuracy of 97.8%. Each model outperformed in terms of training accuracy, and training loss was less than 0.03. The suggested technique extracts layer by layer yielding local and global features during training, which are increasingly abstract as the network convolution layer improves.

**Figure 7:**
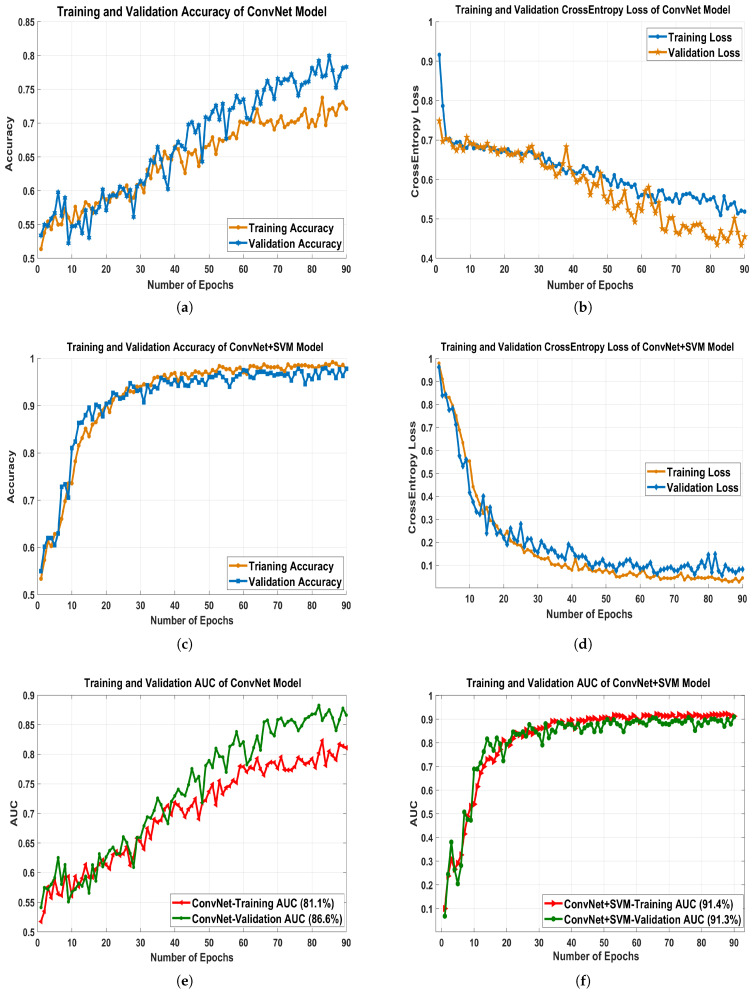
The figures represent the evaluation measures of breast mass detection and classification framework based on the ConvNet architecture. (**a**) Training and validation accuracy; (**b**) training and validation cross-entropy loss; (**e**) AUC, and evaluation measures of breast mass detection and classification framework based on ConvNet+SVM architecture; (**c**) training and validation accuracy; (**d**) training and validation cross-entropy loss; (**f**) AUC.

**Figure 8:**
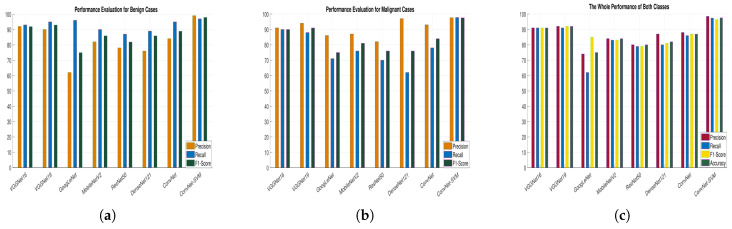
The graphical representation of the results for all comparative experiments: (**a**) Performance analysis for Benign class, (**b**) performance analysis for Malignant class, (**c**) the cumulative performance of both classes.

The proposed architecture’s training time validation accuracies and loss have been presented in Figure 7a–d. Table 4 reveals that, despite having a high overall accuracy rate (ACC), the comparative models have a poor recall rate for the benign and malignant classes. However, a high degree of recall is essential for the classification of medical images. We learned that several deep learning models have a high recall rate. GoogLeNet, MobileNetV2, ResNet50, and DenseNet121 had issues with both classes’ recall, precision, and overall consistency. Four proposed models, the VGGNet16, VGG19, ConvNet, and ConvNet+SVM performed well so we will describe the experimental findings of our projected deep ConvNet+SVM models is depicted in Figure 8a–c. Parameters and time are decreased by using high strides computations in deep convolutional layers. ConvNet+SVM is effectively achieving the highest testing accuracy and the lowest testing loss. The generalization gap (accuracy and losses) between training and validation should be as narrow as possible to avoid overfitting the model.

### 6.3. Performance Measures

**Figure 9:**
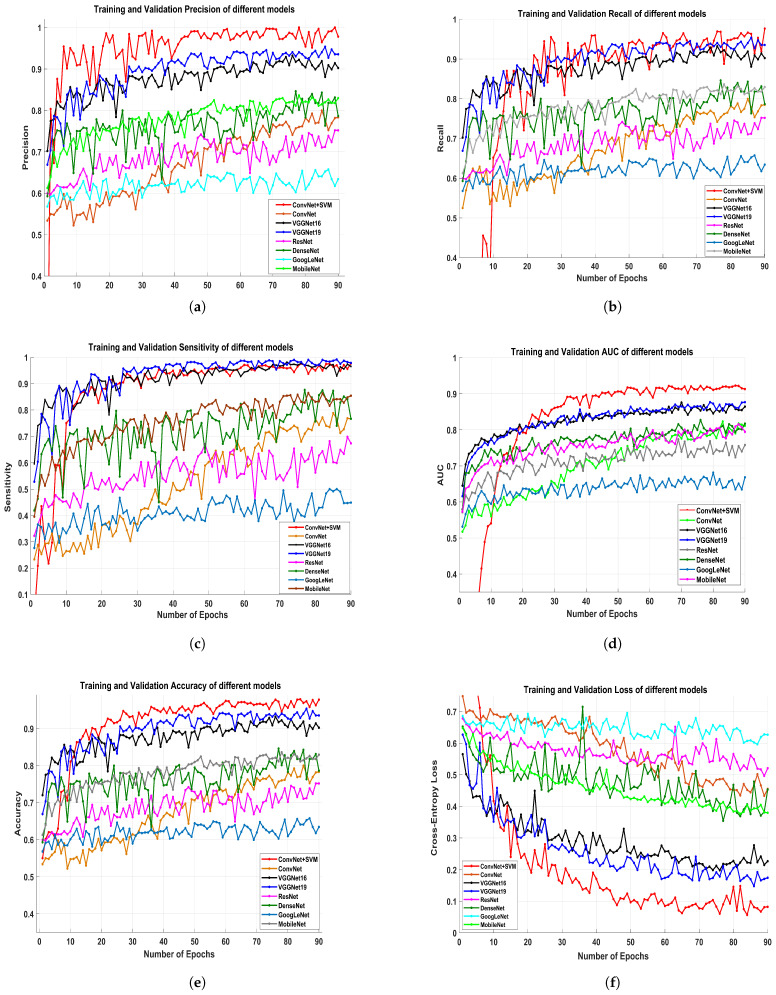
The figures elaborates the performance evaluation of presented models ConvNet+SVM, ConvNet, VGGNet16, VGGNet19, ResNet50, GoogLeNet, DenseNet121, MobileNetV2 in term of the training and validation precision (**a**), recall (**b**), sensitivity (**c**), AUC (**d**), accuracy (**e**), and cross-entropy loss (**f**) against the 90 epochs.

We analyzed the precision rate, recall, F1-scoring, AUC, accuracy, and loss value of each proposed classification technique to determine its efficiency and robustness. Figure 9a–f depicts the training and validation precision, recall, sensitivity, AUC, accuracy and cross-entropy loss of all the proposed pre-trained models. Figure 8a–c demonstrates a more logical comparison of experimental outcomes. The AUC calculated the model’s effectiveness by determining true positives, false positives, true negatives, and false negatives. The ROC and AUC values were obtained for all models, showing that the proposed model performs admirably well in terms of reducing false (positive and negative) rates.

**Figure 10:**
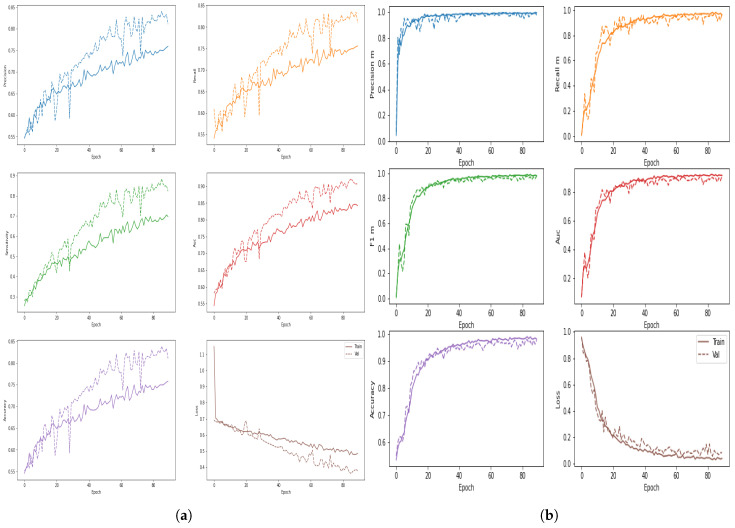
The figures represent the training and validation precision, recall, sensitivity, AUC, accuracy, and cross-entropy loss of (**a**) proposed ConvNet and (**b**) proposed ConvNet+SVM.

The proposed classifier has obtained the highest AUC value of 91.4%. The AUC/ROC curves of the ConvNet+SVM and ConvNet classifiers obtained the maximum training and validation accuracy using the feature set are shown in Figure 7e,f, respectively. In addition, the precision and sensitivity of the ConvNet and ConvNet+SVM classifiers built with the deep feature sets are seen in Figure 10a,b. The findings of all algorithms are summarized in Table 4. After reviewing the results, it was determined that the suggested classifier performed adequately, with precision 97.8%, AUC 91.4%, F1-score 97.06%, and accuracy of 97.8%. The deep learning models’ acquisition of generic image features from ImageNet performed outstanding initialization for the masses or calcification. Misdiagnosis errors for benign images as malignant images are significantly higher than misdiagnosis errors for malignant images as benign images. Thus, the suggested classifier may optimally integrate the predictions from each of the individual architectures. Deep models lead to weak accuracy, poor convergence, and overfitting due to increased layers, increased non-linearities, and a small testing dataset. Learning curves are used in these experiments to assess the behavior of various models during the training and validation phases. These analyses help boost the model’s efficiency by suggesting improvements to the model’s configuration to create a suitable model for breast masses or calcification grading. In general, these experiments aim to create a DCNN model that is highly robust, effective, and reliable in clinical settings. As a result, the model’s precision has to be as inspiring as it is practicable, and the error value as minimal as conceivable.

### 6.4. Comparative Analysis with Conventional Approaches

A comparison of the performance obtained using the implemented architectures and well-known methods are carried out to demonstrate the proposed framework’s strength is given in Table 5. Al-antari et al. [23], introduced a deep learning algorithm to detect, segment, and classify breast mass in mammography. Deepak et al. [47] used pre-trained GoogLeNet architecture for feature learning from MRI images. The author integrated the extracted features using CNN, SVM, and KNN classifiers, achieving 98% accuracy in classification. Khan et al. [48] combined the extracted features by applying DCNNs models VGGNet16, VGGNet19, GoogLeNet, and ResNet50, obtaining an accuracy of 96.6% and an AUC of 93.4%. Rakhlin et al. [49] applied the fusion methods of different DCNN algorithms to determine the sensitivity, AUC for two classes of breast cancer. Ragab et al. [50] presented a novel CAD system based on feature extraction and classification leveraging DL methods to aid radiologists in classifying breast cancer anomalies in mammography. DCNNs were used to extract deep features, which were used to train and evaluate a support vector machine classifier using various kernel functions in the second experiment. The tests were conducted using mammography images from the MIAS dataset and achieved an accuracy of 97.4%. The experiment findings show that the proposed framework attained an accuracy of 97.8% is higher than all the methods.

## 7. Experimental Discussions

The proposed model exhibits high reliability in detecting complex breast masses or calcification, including breast density diversification. In the presented framework, transfer learning has been exploited to overcome the existing systems’ deficiencies in detecting and classifying breast cancer masses. Specific breast masses, such as spiculated and ill-defined lesions, are challenging to detect and label accurately. These breast masses comprising varying types, edges, and dimensions have been appropriately classified in the proposed models. The dense breast’s clinical symptoms are not entirely clear. As a result, it is challenging to distinguish dense lesion characteristics and perform lesion classification correctly. The proposed technique reduces the need for manual mass segmentation by feeding recognized masses or calcification into the classifier directly, decreasing complexities and computing time. The proposed method’s highest test accuracy of 97.8% and AUC score of 91.4% revealed that it should be utilized to aid the clinical decision-making process.

However, the proposed method obtained high performance with limited parameters and significantly enhanced processing time and computation resources. Commonly, thousands of images are used to train deep learning models. One of the current approach’s limitations was the scarcity in the availability of medical imaging data. Training of DCNN models with sparse datasets may overfit and limit the models’ ability to generalize. Different innovative classification models use only one mammogram database for training and validation. The proposed ConvNet+SVM model is capable of identifying mass-affected regions in mammography. The presented models were trained and evaluated on obtained databases, yielding consistent findings demonstrating the robustness in grading under distinct imaging conditions. Parameter optimizing is another contribution to achieve the optimal configuration for the proposed model. For this purpose, various hyperparameters have been manually optimized up to the best classification model. The proposed model has low computational complexity and a fast processing speed, requiring an average testing time of 0.23 to 0.44 s to identify and classify breast masses or calcification, indicating that the model outperforms other conventional DL models. On the other hand, the proposed architecture is known to be reliable and practical for clinical purposes.

## 8. Conclusions and Future Work

Breast cancer is a leading source of morbidity and mortality in worldwide women. It causes patient hospitalizations and ultimately kills a substantial number of patients. According to the WHO, breast cancer is preventable with prompt intervention, timely detection, and treatment. However, access to radiological diagnoses is lacking for the majority of the global population. Additionally, when imaging equipment is available, there is a scarcity of experts who can study mammogram images. This paper proposes the automatic recognition of mammography breast masses or calcification through transfer learning. Deep networks in our approach have more complicated architectures but fewer constraints needing less computational resources but greater consistency. Overfitting problems in mammogram image processing were solved using transfer learning and data augmentation methods which arise when data is inadequate. We modified the architectures of each pre-trained model then fine-tuned each model’s output layers with transfer learning to suit our challenge. We validated our model using 7259 augmented mammography images from MIAS, INbreast, and private databases comprising benign and malignant breast masses or calcification of various sizes, dimensions, and edges.

The various experiments were conducted to determine the model’s robustness and effectiveness. The suggested model has attained 97.8% accuracy, high precision of 97.8%, and AUC of 91.4%, substantiating its efficacy. Despite several methods intended to work with these complex datasets, the proposed methodology yielded excellent results. In the future, we will amend and fine-tune other pre-trained models in the detection stage to increase the system’s performance in the classification stage. Furthermore, using both hand-crafted and CNN features will be enhanced the classification accuracy.

## Figures and Tables

**Table 1:** Augmentation scheme applied in the proposed method.

1	Augmentation Approaches	Setting Values
2	Rotation	45
3	Horizontal Shift	0.15
4	Vertical Shift	0.2
5	Crop and Pad	0.25
6	Zoom Range	0.2
7	Shear	16

**Table 2:** Configuration of model hyper-parameters.

Configuration	Value
Image size	224×224
Epochs	90
Optimization function	Adam
Learning rate	0.001
Batch size	32
Weight decay	0.0001
Activation function	Softmax
Dropout	0.5
Momentum	0.9

**Table 3:** The experimental dataset’s description.

Databases	Benign Images	Malignant Images	Total Images
MIAS	441	357	798
INbreast	1540	861	2401
Private	686	3374	4060
All Datasets	2667	4592	7259
Training Set	2167	4179	6346
Test Set	500	413	913
Total	2667	4592	7259

**Table 4:** Performance analysis of all proposed models in terms of accuracy, precision, recall, F1-score, and area under the curve (AUC) score.

Classifier Name	Precision (%)	Recall (%)	F1_Score (%)	Sensitivity (%)	Training ACC (%)	Training Loss (%)	Validation ACC (%)	Validation Loss (%)	AUC (%)
VGGNet16	90.2	90.2	96.5	93.8	77.1	45.1	90.2	22.3	86.4
VGGNet19	93.5	93.5	97.0	97.0	78.3	43.3	93.5	17.4	87.6
GoogLeNet	63.4	63.4	48.8	53.6	63.4	64.5	63.4	62.6	79.4
MobileNetV2	71.3	71.3	61.6	87.7	71.3	54.0	82.9	37.9	66.8
ResNet50	75.1	75.1	67.3	71.3	67.5	57.7	75.1	52.6	75.8
DenseNet121	78.6	78.0	76.6	86.6	75.1	51.4	72.9	45.2	81.7
Proposed ConvNet	78.3	78.4	76.6	90.0	78.3	45.5	77.1	37.9	87.7
Proposed ConvNet+SVM	97.8	97.7	97.6	97.9	97.7	4.4	97.8	8.2	91.4

**Table 5:** Performance comparison with existing studies.

Author	Methods	Dataset (Nature of Images)	ACC (%)
Khan et al. [48]	Deep features fusion VGG16, ResNet50, GoogLeNet	MIAS, CBIS-DDSM (mammogram)	96.6
Al.antari et al. [23]	YOLO, DCNN, FrCN	INbreast (mammogram)	95.64
Arora et al. [12]	DCNN, AlexNet, VGG16, GoogleNet, ResNet18	DDSM (mammogram)	88.0
Tan et al. [51]	CNN	MIAS (mammogram)	85.5
Vedalankar et al. [52]	DCNN+SVM	MIAS, DDSM (mammogram)	92.0
Albalawi et al. [53]	CNN	MIAS (mammogram)	96.0
Shu et al. [25]	Deep CNN, DenseNet169	INbreast, DDSM (mammogram)	92.4
Agnes et al. [54]	MACNN, Deep CNN	MIAS (mammogram)	96.7
Sha et al. [55]	CNN+SVM	MIAS, DDSM (mammogram)	92.0
Ragab et al. [50]	CNN+SVM	MIAS, (mammogram)	97.4
Proposed model	ConvNet+SVM	MIAS, INbreast, Private	97.8

## Data Availability

The MIAS [44] and INbreast [45] datasets are publicly available and the private [46] data set was collected from a local hospital.
